# Growth Responses of Three European Weeds on Different AMF Species during Early Development

**DOI:** 10.3390/plants11152020

**Published:** 2022-08-03

**Authors:** Verena Säle, Ewald Sieverding, Fritz Oehl

**Affiliations:** 1 Agroscope, Plant Protection Products—Impact and Assessment, Müller-Thurgau-Strasse 29, CH-8820 Wädenswil, Switzerland; fritz.oehl@agroscope.admin.ch; 2 Agroscope, Plant-Soil Interactions, Reckenholzstrasse 191, CH-8046 Zurich, Switzerland; 3 Institute of Agricultural Sciences in the Tropics (Hans-Ruthenberg Institute), University of Hohenheim, Garbenstr. 13, 70599 Stuttgart, Germany; sieverdinge@aol.com

**Keywords:** arbuscular mycorrhizal fungi, mycorrhizal dependency, weed research, growth inhibition, growth response

## Abstract

Arbuscular mycorrhizal fungi (AMF) have multiple functions in agroecosystems and affect many processes below- and aboveground, including plant productivity. Mycorrhizal symbiosis is not necessarily beneficial for the host plant and the growth response can be not only positive but also neutral or negative. Among other factors, the responsiveness of plants to AMF depends on the plant-fungus combination. To find out whether the AMF species or isolate is a decisive factor for growth responses of weeds, 44 AMF isolates were tested in a pot experiment for their effects on three agricultural weeds: *Echinochloa crus-galli*, *Solanum nigrum* and *Papaver rhoeas*. The 44 isolates cover 18 AMF species from 13 genera and all 5 orders of the Glomeromycota. The aboveground biomass of the weeds was determined after different times of growth of each weed. In most cases, the effects of AMF isolates on weed growth were negative or neutral. We conclude that some weed species do not benefit from AMF in terms of growth. AMF species can even cause negative growth responses, an effect that may be of practical interest for organic farming where the aim is to obtain a high diversity and concentration of native AMF for the benefit of the cultivated crops without increasing the labor for mechanical weeding.

## 1. Introduction

Arbuscular mycorrhizal fungi (AMF) are obligate root endophytes which rely on getting carbohydrates from plants. In return, the fungi provide multiple ecosystem services: AMF facilitate nutrient acquisition and uptake for plants, especially of phosphorus. Furthermore, they enhance resistance against drought and root pathogens [1,2]. In addition, they can have positive effects on soil aggregation, and they prevent soil and nutrient losses [1,2]. AMF can change the diversity and productivity of plant communities [3] and alter plant competition [4,5]. Several studies also indicate that AMF may affect the composition and functioning of weed communities [6].

Weeds are considered to play an important role for AMF diversity in agronomic crops [7,8], and in particular, the negative effects of eliminating weeds through herbicides applications have been shown. Though the positive effects of weeds for AMF communities have been investigated, there are only a few studies on the effects of AMF on weed growth. Some weed species have been reported to respond negatively to AMF, in line with studies showing that plant growth responses to AMF may be mutualistic, neutral or antagonistic—depending on the plant-AMF combination and on environmental conditions [9,10,11,12].

Because of the negative reaction of some weeds towards AMF, it has been suggested that AMF may be useful for integrated weed control [6,13]. When crops get better access to nutrients than weeds, they can get a growth advantage and be more competitive, which, thus, facilitates weed control [14,15]. Differences in the mycorrhizal dependency between crops and weeds can change the competitiveness of these plants [16].

Our objective was to gain more insight into species-specific effects between AMF and host weeds. Differences in growth response between AMF species or even between isolates have been shown for several crops and other plants [10,17,18,19,20]. However, investigations specifically on agriculturally relevant weeds are mostly limited to a few AMF species; screenings encompassing many species and isolates from different families are missing. Therefore, we inoculated plants of *Echinochloa crus-galli*, *Solanum nigrum* and *Papaver rhoeas* with a wide range of AMF isolates and AMF species of all higher taxa levels (class and order) and measured the plant growth response. We hypothesised that the response of weeds depends on the applied AMF taxum or isolate and on the weed species.

## 2. Results

### 2.1. Echinochloa crus-galli

**Figure 1:**
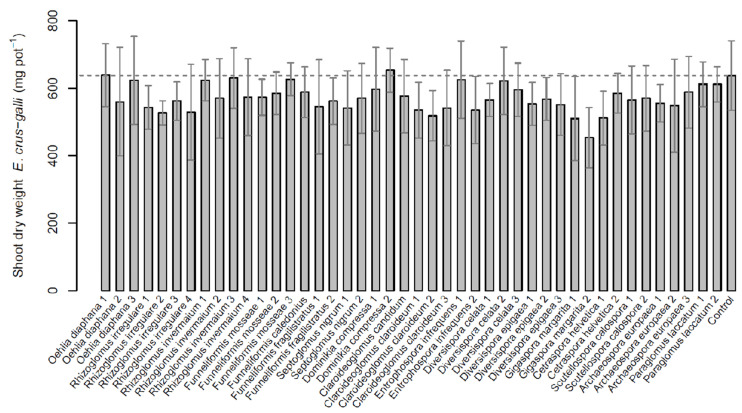
Aboveground dry mass of *Echinochloa crus-galli* inoculated with 44 different isolates of arbuscular mycorrhizal fungi (AMF) and one non-mycorrhizal control. Data are reported as means (n = 6) and their standard deviations. No significant differences among AMF isolates or the control treatment were detected via Kruskal–Wallis test (*p* < 0.05).

The mean shoot biomass of Echinochloa crus-galli of the non-mycorrhizal control treatment was 638 mg per pot. Biomasses of the AMF treatments were not significantly different compared to the control or between isolates (*χ*2 = 52.52, df = 44, *p* = 0.18). However, the biomass of AMF-inoculated plants tended to be lower (Figure 1). Only two isolates led to the same (Oehlia diaphana, O.dia1) or a slightly higher biomass (*Dominikia compressa*, Do.com2; 653 mg per pot) than the control. Inoculation with *Gigaspora margarita* (G.mar2) had the lowest biomass (453 mg), followed by *Diversispora epigeae* (Di.epi1, 507 mg) and *G. margarita* (G.mar1, 510 mg).

On the species level, pairwise comparisons showed that *G. margarita* resulted in a significantly lower biomass of *E. crus-galli* than some of the other species and than the control treatment, while *D. compressa* was significantly higher than *Rhizoglomus irregulare* and *Claroideoglomus claroideum* (Table 1). No significant differences were detected for other species.

Mycorrhizal dependency ranged from 2% (Do.com2) to −46% (G.mar2; Table 2), which reflects the results for the biomass. No significant differences were detected. 

### 2.2. Solanum nigrum

**Figure 2:**
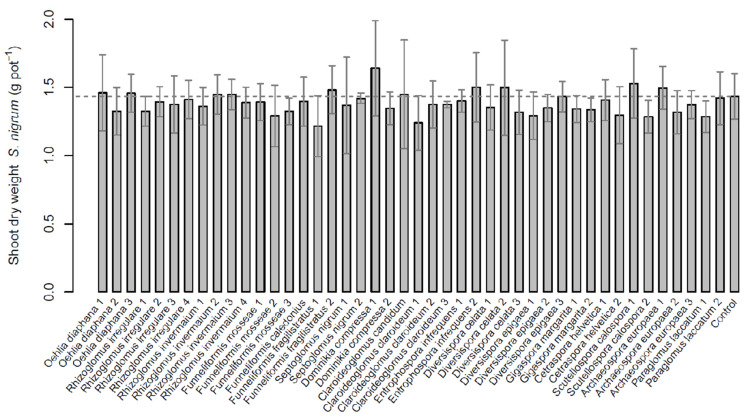
Aboveground dry mass of *Solanum nigrum* inoculated with 44 different isolates of arbuscular mycorrhizal fungi (AMF) and one non-mycorrhizal control. Data are reported as means (n = 6) and their standard deviations. No significant differences among AMF isolates or the control treatment were detected via Kruskal–Wallis test (*p* < 0.05).

Also for *Solanum nigrum* no significant differences in shoot growth response to AMF inoculation were detected, neither between AMF isolates and the control, nor between different AMF isolates (*χ*^2^ = 44.59, df = 44, *p* = 0.45). The mean biomass per pot of *S. nigrum* ranged from 1215 mg for isolate *Funneliforme fragilistratus* (F.fra1) to 1641 mg for *D. compressa* (Do.com1). The control treatment was in between with 1433 mg (Figure 2).

Comparisons on the species level also could not detect significant differences (*χ*^2^ = 9.39, df = 18, *p* = 0.95). Mycorrhizal dependency was lowest for *F. fragilistratus* (F.fra1, −22%) and highest for *D. compressa* (Do.com2, 10%; Table 2). However, there were no significant differences.

### 2.3. Papaver rhoeas

**Figure 3:**
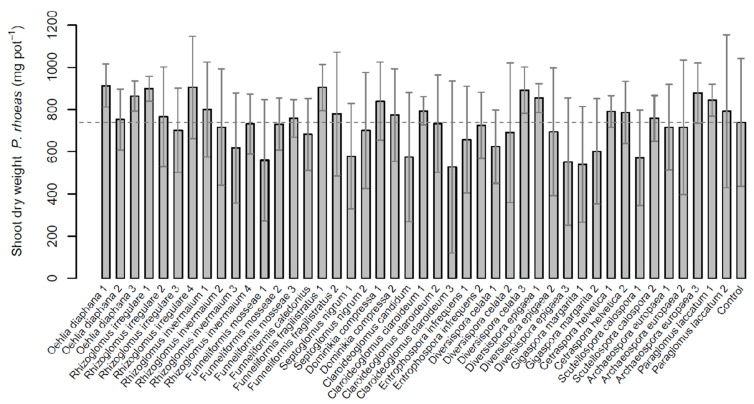
Aboveground dry mass of *Papaver rhoeas* inoculated with 44 different isolates of arbuscular mycorrhizal fungi (AMF) and one non-mycorrhizal control. Data are reported as means (n = 6) and their standard deviations. No significant differences among AMF isolates or the control treatment were detected via Conover’s all-pairs comparisons (*p* < 0.05).

The shoot biomass of the *Papaver rhoeas* control treatment was 739 mg per pot. Half of the AMF isolates increased the biomass above the control, with the highest biomasses in *F. fragilistratus* (F.fra1, 905 mg), *R. irregulare* (R.irr4, 905 mg) and *O. diaphana* (O.dia1, 914 mg). The lowest growth responses were found for *C. etunicatum* (Cl.etu) and *G. margarita* (G.mar1) with 528 mg and 540 mg, respectively (Figure 3). Although the emergence of *P. rhoeas* seeds were very unequal and the variation of plant growth was high both between and within treatments, the Kruskal–Wallis test revealed a significant influence of the factor inoculation on plant biomass (*χ*^2^ = 72.99, df = 44, *p* < 0.05). However, the post-hoc test (with adjusted *p*-values) could not detect any significant differences between AMF isolates and the control treatment. This was the same case for the mycorrhizal dependency (Table 2). On the species level, inoculation with *G. margarita* led to a significant lower biomass than *O. diaphana* and *F. fragilistratus*.

## 3. Discussion

In the current study, plant growth of the three weed species *S. nigrum*, *E. crus-galli* and *P. rhoeas* was significantly influenced by an inoculation with only a few of the 44 AMF isolates. There are some tendencies: both *Gigaspora margarita* isolates suppressed the biomass of all the weed species, while *Oehlia diaphana* (especially the isolates O.dia1 and O.dia3) seemed to be more beneficial for the weeds.

Almost all of the AMF isolates in this experiment were formerly tested on leek plants. The leek plants were cultivated at the same time and under the same conditions as *E. crus-galli* and *S. nigrum*. In this other experiment, it was shown that that the majority of the isolates gave a positive growth response to the plants [21] (Table 2). This indicates that the isolates were vital and have the ability to influence plant growth. 

In general, the responsiveness of plants to AMF can be positive, neutral or negative [9,10]. Studies of Vatovec, et al. [22] that were performed on weeds and ruderal plants also show this variation in growth response, ranging from enhanced growth to growth depression. Veiga, et al. [23] found that several weeds (including *E. crus-galli* and *S. nigrum*) inoculated with R. intraradices or with a mixture of *R. intraradices*, *C. claroideum* and *F. mosseae* were repressed or did not benefit growth significantly. Rashidi, et al. [24] reported similar effects: *S. nigrum* in monoculture was suppressed by *F. mosseae* and *R. intraradices*; when *S. nigrum* was grown together with *Phaseolus vulgaris*, the negative effects on *S. nigrum* were even more pronounced. In addition, suppression of weeds in the field was found when AMF was present: Jordan and Huerd [6] detected higher weed densities in the field when the mycorrhizal symbiosis was repressed by the fungicide Benomyl. 

Improved growth and seed production of weeds and offsprings due to an inoculation with *R. intraradices* was reported [25,26,27,28,29]. A positive or neutral response of six ruderal plant species was also found by Del Fabbro and Prati [30]. Comparing wild plants and cultivated ones, Koide, et al. [26] showed that wild plants might benefit less: although *R. intraradices* improved the growth of cultivated as well as of wild oats, the benefit in reproduction was larger in cultivated oats and the duration of flowering and lifespan were negatively affected in wild oats.

The same weed species can respond differently to AMF fungi depending on the growth condition. For instance, Veiga, et al. [23] found that *E. crus-galli* and *S. nigrum* inoculated with *R. intraradices* were repressed significantly. However, Rinaudo, et al. [31] showed that AMF reduced the biomass of *E. crus-galli* only, when it was grown together with sunflower, but not when the weed was grown alone. For *S. nigrum*, Vatovec, et al. [22] found no significant effect on growth, although under certain environmental conditions, the growth response tended to be negative, which is similar to our results. On the other hand, different isolates from the same AMF species can lead to contrasting results regarding plant growth response [10,17,18,19,20]. In our study, these effects were less pronounced, i.e., there were also differences among different isolates of the same species, but they were not significant.

Wild plants are more adapted to unfavorable growth conditions, such as low soil fertility, than cultivated plants, and therefore, they may respond differently to AMF [30,32]. Factors such as root architecture may also affect the mycorrhizal growth response: Yang et al. (2015) found that plants with taproots showed a higher growth response than those with fibrous root systems. In our study however, we used weeds with taproots (*S. nigrum* and *P. rhoeas*) as well as a weed with fibrous roots (*E. crus-galli*), and consistent positive growth effects due to AMF isolates on the broad leaf weeds were not found. A meta-analysis of Li [13] suggests that weak host weeds (i.e., with low root colonization potential due to AMF) are more repressed by AMF than strong host weeds (high root colonisation potential). 

El Omari and El Ghachtouli [33] name different mechanisms underlying the repression of weeds by AMF. Firstly, there can be direct effects as AMF act like weak parasites for weeds, or plants invest energy in a kind of self defense against root colonization by AMF so that plant development suffers at an early stage. The developmental stage of the host can have a direct effect, i.e., young plants invest in their mycorrhizal network but receive the benefits from the symbiosis later. Second, indirect effects via the interaction of AMF, weeds and crops occur where the nutrient fluxes can be disadvantageous for weeds, so that the weeds are suppressed in growth.

According to Johnson, et al. [9], AMF is not beneficial when the net costs exceed the net benefits of plants. The lack of benefit can be caused by the high carbon demand of AMF or due to the replacing of the direct P uptake via plant roots by the less efficient fungal pathway [1,9]. The efficiency of an AM association depends on the AM fungus, the host plant and the growth conditions [11,34]. The influence of the identity of the fungus and host is here especially confirmed by the fact that the same AMF isolates that were applied in this weed experiment gave different results in another test where leek was used as the host plant [21]. This other study showed that the AMF isolates enhanced leek biomass depending on the AMF species and the clade they belong to [21]. Growth responses of plants to AMF depend on the situation under which they grow, which was called “context-dependency of AMF” by van der Heijden, et al. [2].

In this study, we exclusively focused on the biomass of weeds and not on any other ecosystem functions of AMF. For instance, AMF can prevent nutrient losses [2,35,36], even when there is no growth response [37], they improve soil structure via soil aggregating [38] and they provide many other benefits such as increased nutrient-use efficiency, litter decomposition or resistance to heavy metals [2]. Furthermore, in this study we investigated monocultures only. Rinaudo, et al. [31] and Veiga, et al. [23] demonstrated that in the presence of a crop, AMF weeds were more suppressed than when grown alone. These interactions of weeds and crops were not assessed here but should be considered in further investigations.

## 4. Conclusions

All three of the European weeds we assessed, depend to a low level on AMF. In most cases, the shoot growth responses of weeds were negative as a result of inoculation with Glomeromycotean species. This study emphasizes, together with other experiments [21], the importance of AMF and host plant identity, as not every plant-fungus combination results in the same outcome regarding biomass production. From a practical point of view—and in particular in the context of organic farming—this is of interest: When crop plants are more responsive towards AMF than weeds, this could be advantageous for weed control. However, a negative or low response of a weed to colonization with an AMF species may be context dependent, i.e., dependent upon the circumstances under which the weeds get colonized by an AMF species. This must be considered when discussing the role of AMF for weed growth inhibition or crop yield improvement under natural conditions.

## 5. Materials and Methods

### 5.1. Establishment of AMF Isolate Cultures

In the experiment, 44 isolates from the Swiss collection for arbuscular mycorrhizal fungi (SAF) were used. The isolates were derived in Switzerland from agricultural soils with different farming practices, except the two *Scutellospora calospora* isolates (Sc.cal1 and Sc.cal2), which originated from soils in Germany. After propagation from soil and isolation from AMF trap cultures, single or multi spore cultures were established for each AMF species and propagated identically for 12 months in the greenhouse using *Hieracium pilosella* as host plant. For a detailed description of the propagation process, see Oehl, et al. [39], Oehl, et al. [40], Tchabi, et al. [20] and Säle, et al. [21]. The substrates with the propagated spores were dried and used as inocula for the weed plants in the present experiment. The isolates that were selected for this experiment comprised 18 species from 13 genera, 8 families and all 5 orders (Table 3). A molecular characterization of the isolates is given in Säle, et al. [21]. The large majority of the AMF isolates and all species in this study were also tested in a screening using leek as host plant [21].

### 5.2. Experimental Setup

A pot experiment was established, with three mycorrhizal weed species each tested with the 44 AMF isolates (see above) and one non-mycorrhizal control. As weed species we selected *Echinochloa crus-galli* (gramineous summer weed), *Solanum nigrum* (herbaceous summer weed) and *Papaver rhoeas* (herbaceous winter weed). Seeds were obtained from Herbiseed (Twyford, UK). Each treatment was replicated six times, resulting in 810 pots for the whole experiment. For the substrate, Loess sub soil and quartz sand were autoclaved at 121 °C for 90 min and mixed to the equal proportion of weight. Measurements of soil parameters were done according to standard methods in the laboratory of F.M. Balzer, Wetter-Amönau, Germany (see Oehl, et al. [46]). The following chemical properties of the substrate were quantified: pH (H_2_O) = 6.0, Corg = 1.4 g kg^−1^, *p* = 8.3 mg kg^−1^, K = 31.5 mg kg^−1^, Ca = 910 mg kg^−1^, Mg = 149 mg kg^−1^. P, K and Mg were extracted with double lactate, Ca with HCl and H_2_SO_4_. For the treatments with *Solanum nigrum* and *Echinochloa crus-galli* pots were filled with 500 g substrate and watered to 100% water capacity. In the middle of the pots, 5 mL AMF inoculum were placed and afterwards covered with a thin layer of substrate. For the nonmycorrhizal control, 5 mL of the same substrate, similar to the AMF inoculum but not containing vital AMF propagules, was used identically for 12 months as substrate in the greenhouse, but on non-mycorrhizal *Hieracium pilosella* as host plant. Fifteen seeds were placed above the inocula and covered with quartz sand. After emergence, seedlings were thinned to two plants per pot.

The screening experiment with *Solanum nigrum* and *Echinochloa crus-galli* started in early summer (June 2012). No additional lightening or heating was applied during the experiment in the greenhouse, and the plants were kept as close as possible to ambient temperature and light conditions with average temperatures of 25 °C during the day and 18 °C at night. During the experiment, weeds were fertilized with 50 mg N, 20 mg P and 50 mg K per kg substrate in the form of a solution of NH_4_NO_3_, KH_2_PO_4_ and K_2_SO. Eight weeks after emergence, the aboveground biomass was harvested. For *Papaver rhoeas*, pots were set up in the same way as for the two other weeds, but the sowing time was in autumn (November 2012). To enable vernalization, no additional lightning or heating was applied also for *P. rhoeas*. Nevertheless, during wintertime, the temperature was never below 3 °C. *Papaver rhoeas* received the following fertilization: 50 mg N, 20 mg P and 50 mg K per kg substrate in early spring, the same amount again two months later and 50 mg N only before flowering. Harvest of *P. rhoeas* was after 5 months, when inflorescences of plants emerged (BBCH development stage 5). For each plant species, pots were regularly completely randomized. Biological plant protection with predators was carried out with *Hypoaspis miles* and *Phytoseiulus persimilis* against spider mites and with *Amblyseius swirskii* against thrips and whitefly. Aboveground biomass of weeds was oven dried for 48 h at 60 °C and weighed afterwards.

### 5.3. Root Colonization

AMF root colonization was not measured quantitatively. However, for each weed species six random samples that were taken from the treatments with *Glomus* and *Rhizoglomus* species (intensive root colonizing species) gave the proof that AMF were present in the roots, i.e., the inoculation was successful. To check whether roots were colonized with AMF, roots were stained with trypan blue according to the method of Koske and Gemma [47]. For this purpose, roots were washed, cut into small pieces, cleared with 10% KOH and stained with 0.05% trypan blue in lactoglycerol. Stained roots were placed on slides and inspected under the microscope.

In another study with leek plants, all treatments were checked for root colonization [21]. The isolates that were used in the present experiment are from the same batch, so we can be sure that the spores were vital. 

### 5.4. Statistical Analyses

Mycorrhizal dependency was calculated according to Plenchette, et al. [48]:

$$\mathrm{mycorrhizal}\;\mathrm{dependency}\;\left(\%\right)=\frac{\mathrm{biomass}\;\mathrm{mycorrhizal}\;\mathrm{plant}-\mathrm{biomass}\;\mathrm{control}}{\mathrm{biomass}\;\mathrm{mycorrhizal}\;\mathrm{plant}}\times 100$$

For each weed species, the effect of an inoculation with different AMF isolates on weed biomass was analyzed using the non-parametric Kruskal–Wallis rank sum test, as data showed no normal distribution of residuals and no homogeneity of variances. For post-hoc comparisons, Conover’s test for all-pairs comparisons was applied. In order to correct for multiple testing, *p*-values were adjusted according to the Benjamini–Hochberg procedure. All significance levels were set at *p* < 0.05. Figures show estimates of the means and error bars for the standard deviations. The statistical analyses and graphs were carried out with the software R 4.0.5 [49] using the packages stats, graphics and PMCMRplus [50].

## Figures and Tables

**Table 1:** Significant differences between different AMF species in plant biomass development of *E. crus-galli* obtained via all-pairs comparisons after Kruskal–Wallis rank sum test (non significant differences between AMF species are not shown).

	*Gigaspora margarita*	*Dominikia compressa*
Control	*p* = 0.049	
*Oehlia diaphana*	*p* = 0.040	
*Rhizoglomus invermaium*	*p* = 0.040	
*Funneliformis mosseae*	*p* = 0.049	
*Dominikia compressa*	*p* = 0.025	
*Paraglomus laccatum*	*p* = 0.043	
*Rhizoglomus irregulare*		*p* = 0.049
*Claroideoglomus claroideum*		*p* = 0.049

**Table 2:** Mycorrrhizal dependency (in %) of the weeds *E. crus-galli*, *S. nigrum* and *P. rhoeas* in comparison to leek plants adapted with permission from a study from Säle, et al. [21]. Data show means of six replicates ± SD; n.a. = not available.

Isolate	Mycorrhizal Dependency (%)			
	*E. crus-galli*	*Solanum nigrum*	*Papaver rhoeas*	Leek
O.dia1	−2 ± 16	−1 ± 18	18 ± 9	168 ± 30
O.dia2	−23 ± 41	−10 ± 15	−2 ± 25	160 ± 23
O.dia3	−7 ± 27	1 ± 10	14 ± 7	153 ± 26
R.irr1	−19 ± 17	−9 ± 8	17 ± 5	148 ± 33
R.irr2	−21 ± 8	−3 ± 8	−7 ± 42	124 ± 29
R.irr3	−14 ± 12	−7 ± 18	−16 ± 47	155 ± 36
R.irr4	−28 ± 34	−2 ± 11	14 ± 19	148 ± 32
R.inv1	−3 ± 11	−6 ± 12	−4 ± 51	135 ± 12
R.inv2	−17 ± 31	0 ± 9	−44 ± 132	174 ± 28
R.inv3	−3 ± 15	0 ± 8	−40 ± 61	152 ± 24
R.inv4	−15 ± 24	−4 ± 8	−5 ± 26	163 ± 17
F.mos1	−12 ± 10	−4 ± 11	−141 ± 287	134 ± 21
F.mos2	−10 ± 13	−14 ± 21	−4 ± 20	121 ± 27
F.mos3	−2 ± 9	−9 ± 8	1 ± 12	129 ± 25
F.cal	−10 ± 14	−4 ± 13	−13 ± 25	143 ± 18
F.fra1	−24 ± 32	−22 ± 23	17 ± 10	94 ± 16
F.fra2	−15 ± 15	2 ± 11	−26 ± 103	100 ± 23
Se.nig1	−24 ± 35	−9 ± 22	−66 ± 110	107 ± 29
Se.nig2	−15 ± 22	−1 ± 3	−45 ± 127	98 ± 32
Do.com1	−12 ± 32	10 ± 18	7 ± 28	93 ± 11
Do.com2	2 ± 9	−7 ± 10	−7 ± 49	84 ± 32
Cl.can	−15 ± 26	−4 ± 24	−166 ± 366	104 ± 29
Cl.cla1	−22 ± 21	−18 ± 18	6 ± 8	123 ± 23
Cl.cla2	−25 ± 21	−6 ± 13	−10 ± 37	127 ± 19
Cl.cla3	−24 ± 37	−4 ± 2	−467 ± 799	125 ± 21
E.inf1	−5 ± 19	−3 ± 6	−59 ± 147	76 ± 16
E.inf2	−24 ± 32	2 ± 14	−6 ± 23	124 ± 27
Di.cel1	−14 ± 10	−7 ± 15	−27 ± 38	n.a.
Di.cel2	−5 ± 17	1 ± 20	−162 ± 413	111 ± 28
Di.cel3	−9 ± 14	−10 ± 15	16 ± 12	110 ± 40
Di.epi1	−16 ± 13	−12 ± 15	13 ± 7	101 ± 31
Di.epi2	−13 ± 12	−7 ± 7	−94 ± 249	119 ± 29
Di.epi3	−18 ± 20	−1 ± 8	−128 ± 219	n.a.
G.mar1	−35 ± 50	−7 ± 8	−284 ± 643	79 ± 16
G.mar2	−46 ± 30	−8 ± 7	−49 ± 76	89 ± 27
Ce.hel1	−27 ± 20	−3 ± 13	6 ± 9	86 ± 13
Ce.hel2	−10 ± 11	−13 ± 18	2 ± 23	n.a.
Sc.cal1	−16 ± 19	4 ± 16	−83 ± 168	n.a.
Sc.cal2	−15 ± 20	−12 ± 12	1 ± 14	n.a.
A.eur1	−16 ± 11	3 ± 10	−15 ± 53	93 ± 20
A.eur2	−24 ± 37	−10 ± 14	−95 ± 257	77 ± 17
A.eur3	−12 ± 28	−5 ± 8	13 ± 17	n.a.
P.lac1	−5 ± 12	−12 ± 10	12 ± 7	67 ± 18
P.lac2	−5 ± 10	−2 ± 13	−85 ± 254	n.a.

**Table 3:** List of AMF isolates of this study together with reference collection numbers (SAF = Swiss collection of arbuscular mycorrhizal fungi; original accession number) and information on the original isolation sites of the AMF isolates. Nomenclature is according to Oehl, et al. [41] and Oehl, et al. [42], updated in Baltruschat, et al. [43] and Wijayawardene, et al. [44]. Some isolates may be named differently by other authors (e.g., [44,45]).

AMF Isolate	Species	Family	Order	SAF Accession	Original Accession	Land Use at Origin Site	Vegetation at Origin Site	Soil pH	Soil Type at Origin
O.dia1	*Oehlia diaphana*	Glomeraceae	Glomerales	SAF106	11-FO106	arable field	winter wheat	5.3	Eutric Cambisol
O.dia2	*Oehlia diaphana*			SAF107	11-FO290	arable field	winter barley	5.6	Eutric Cambisol
O.dia3	*Oehlia diaphana*			SAF108	11-FO292	arable field	winter barley	5.6	Eutric Cambisol
R.irr1	*Rhizoglomus irregulare*			SAF130	11-FO113	arable field	winter wheat	5.3	Haplic Luvisol
R.irr2	*Rhizoglomus irregulare*			SAF131	11-FO190	arable field	winter wheat	7.6	Vertic Cambisol
R.irr3	*Rhizoglomus irregulare*			SAF170	11-FO420	permanent grassland	grassland	5.5	Eutric Cambisol
R.irr4	*Rhizoglomus irregulare*			SAF96	11-FO181	arable field	winter wheat	7.6	Vertic Cambisol
R.inv1	*Rhizoglomus invermaium*			SAF205	11-FO84	arable field	grass–clover	7.1	Eutric Cambisol
R.inv2	*Rhizoglomus invermaium*			SAF206	11-FO424	permanent grassland	grassland	5.5	Eutric Cambisol
R.inv3	*Rhizoglomus invermaium*			SAF207	11-FO432	permanent grassland	grassland	5.8	Eutric Cambisol
R.inv4	*Rhizoglomus invermaium*			SAF147	11-FO336	permanent grassland	grassland	5.8	Eutric Cambisol
F.mos1	*Funneliformis mosseae*			SAF87	11-FO85	arable field	grass–clover	7.1	Haplic Luvisol
F.mos2	*Funneliformis mosseae*			SAF139	11-FO239	arable field	winter barley	5.6	Haplic Luvisol
F.mos3	*Funneliformis mosseae*			SAF160	11-FO418	permanent grassland	grassland	5.5	Eutric Cambisol
F.cal	*Funneliformis caledonius*			SAF111	11-FO269	arable field	winter barley	5.6	Haplic Luvisol
F.fra1	*Funneliformis fragilistratus*			SAF109	11-FO185	arable field	winter wheat	7.6	Vertic Cambisol
F.fra2	*Funneliformis fragilistratus*			SAF110	11-FO193	arable field	winter wheat	7.6	Vertic Cambisol
Se.nig1	*Septoglomus nigrum*			SAF86	11-FO61	permanent grassland	grassland	5.7	Haplic Luvisol
Se.nig2	*Septoglomus nigrum*			SAF175	11-FO471	arable field	winter barley	7.1	Eutric Cambisol
Do.com1	*Dominikia compressa*			SAF145	11-FO332	permanent grassland	grassland	5.8	Eutric Cambisol
Do.com2	*Dominikia compressa*			SAF203	11-FO352	permanent grassland	grassland	5.8	Eutric Cambisol
Cl.can	*Claroideoglomus candidum*	Entrophosporaceae		SAF112	11-FO411	permanent grassland	grassland	5.5	Eutric Cambisol
Cl.cla1	*Claroideoglomus claroideum*			SAF92	11-FO55	permanent grassland	grassland	5.7	Haplic Luvisol
Cl.cla2	*Claroideoglomus claroideum*			SAF181	11-FO94	permanent grassland	grassland	7.1	Haplic Luvisol
Cl.cla3	*Claroideoglomus claroideum*			SAF166	11-FO370	arable field	grass–clover	6.2	Haplic Luvisol
E.inf1	*Entrophospora infrequens*			SAF209	11-FO321	arable field	grass–clover	6.2	Eutric Cambisol
E.inf2	*Entrophospora infrequens*			SAF210	11-FO313	arable field	grass–clover	6.2	Eutric Cambisol
Di.cel1	*Diversispora celata*	Diversisporaceae	Diversisporales	SAF5	HG-234	permanent grassland	grassland	7.0	Haplic Luvisol
Di.cel2	*Diversispora celata*			SAF151	11-FO387	permanent grassland	grassland	5.3	Haplic Luvisol
Di.cel3	*Diversispora celata*			SAF152	11-FO403	permanent grassland	grassland	5.5	Haplic Luvisol
Di.epi1	*Diversispora epigaea*			SAF118	11-FO459	arable field	winter barley	7.1	Eutric Cambisol
Di.epi2	*Diversispora epigaea*			SAF128	11-FO338	permanent grassland	grassland	5.8	Eutric Cambisol
Di.epi3	*Diversispora epigaea*			SAF129	11-FO460	arable field	winter barley	7.1	Eutric Cambisol
G.mar1	*Gigaspora margarita*	Gigasporaceae	Gigasporales	SAF14-1	JJ-4	arable field	winter wheat	6.2	Haplic Luvisol
G.mar2	*Gigaspora margarita*			SAF14-2	JJ-4	arable field	winter wheat	6.2	Haplic Luvisol
Ce.hel1	*Cetraspora helvetica*	Racocetraceae		SAF15-1	JJ17/19	arable field	winter wheat	6.2	Haplic Luvisol
Ce.hel2	*Cetraspora helvetica*			SAF15-2	JJ17/19	arable field	winter wheat	6.2	Haplic Luvisol
Sc.cal1	*Scutellospora calospora*	Scutellosporaceae		SAF202-1	01-FO30	vineyard	grapevine	7.7	Eutric Cambisol
Sc.cal2	*Scutellospora calospora*			SAF202-2	01-FO30	vineyard	grapevine	7.7	Eutric Cambisol
A.eur1	*Archaeospora europaea*	Archaeosporaceae	Archaeopsporales	SAF113	11-FO107	arable field	winter wheat	5.3	Eutric Cambisol
A.eur2	*Archaeospora europaea*			SAF114	11-FO126	arable field	winter wheat	7.6	Vertic Cambisol
A.eur3	*Archaeospora europaea*			SAF115	11-FO345	permanent grassland	grassland	5.8	Eutric Cambisol
P.lac1	*Paraglomus laccatum*	Paraglomeraceae	Paraglomerales	SAF56-1	BEG21	permanent grassland	grassland	7.7	Calcaric Leptosol
P.lac2	*Paraglomus laccatum*			SAF56-2	BEG21	permanent grassland	grassland	7.7	Calcaric Leptosol

## Data Availability

Data from this study are available upon request from the corresponding author.
